# Optimization of *E. coli* Inactivation by Benzalkonium Chloride Reveals the Importance of Quantifying the Inoculum Effect on Chemical Disinfection

**DOI:** 10.3389/fmicb.2018.01259

**Published:** 2018-06-26

**Authors:** Míriam R. García, Marta L. Cabo

**Affiliations:** ^1^Bioprocess Engineering Group, IIM-CSIC Spanish National Research Council, Vigo, Spain; ^2^Microbiology Group, IIM-CSIC Spanish National Research Council, Vigo, Spain

**Keywords:** benzalkonium chloride (alkyldimethylbenzylammonium chloride), *Escherichia coli*, disinfection, inactivation, kinetic modeling, inoculum effect

## Abstract

Optimal disinfection protocols are fundamental to minimize bacterial resistance to the compound applied, or cross-resistance to other antimicrobials such as antibiotics. The objective is twofold: guarantee safe levels of pathogens and minimize the excess of disinfectant after a treatment. In this work, the disinfectant dose is optimized based on a mathematical model. The model explains and predicts the interplay between disinfectant and pathogen at different initial microbial densities (inocula) and dose concentrations. The study focuses on the disinfection of *Escherichia coli* with benzalkonium chloride, the most common quaternary ammonium compound. Interestingly, the specific benzalkonium chloride uptake (mean uptake per cell) decreases exponentially when the inoculum concentration increases. As a consequence, the optimal disinfectant dose increases exponentially with the initial bacterial concentration.

## 1. Introduction

Quaternary ammonium compounds (QACs) are chemicals produced at high volumes with low toxicity that may induce resistance to disinfectants or cross-resistance to other antimicrobials (Langsrud et al., 2003; Tezel and Pavlostathis, 2015). They are widely used in medical-related facilities, and in the food and pharmaceutical industries.

QACs differ from common disinfectants in water treatments as they are not chemically transformed during their application and may be released and diluted in the environment. Most QACs are only degraded under aerobic conditions by bacterial species in the genera of *Xanthomonas, Aeromonas*, and *Pseudomonas*. The impact of the degraders is still a matter of controversy. Tezel and Pavlostathis (2011) claim that this biodegradation creates sub-inhibitory concentrations in environmental media such as surface water and soil where susceptible species may develop bacterial resistance. That effect is not significant for some authors that restrict the environmental consequences to a change in bacterial tolerance to antimicrobials but not to resistance (Gerba, 2015). In any case, QACs degraders proliferate and may eventually cause public health problems (Tezel and Pavlostathis, 2015).

Optimal disinfection protocols are critical to minimizing QACs excess after a treatment while assuring safety levels of pathogens. Disinfectant dose concentrations smaller than the optimum are insufficient to achieve the necessary inactivation level and may induce resistance to the disinfectant applied. Larger doses guarantee that most pathogens are inactivated but may induce resistance in surrounding areas where disinfection concentration is lower because of partial coverage (Holah et al., 2002). Moreover, active chemicals may end up in the environment after the treatment and induce cross-resistance to other antimicrobials, including relevant antibiotics. In addition to reducing environmental impact, optimal doses contribute to minimizing the cost of the disinfection treatment.

Nowadays, QACs disinfection is mostly based on minimum inhibitory numbers without any advanced optimization due to the lack of appropriate models. Let us consider for example alkyldimethylbenzylammonium chloride, commonly known as benzalkonium chloride (BAC). This compound is a widely used “over-the-counter” surface disinfectant that may increase tolerance to antibiotics in *E. coli* (Bore et al., 2007). Kinetic models are scarce and mostly limited to time-kill curves without considering the concentration of BAC during the treatment (Ioannou et al., 2007). The few exceptions considering sophisticated models are not focused on the disinfection itself, but on subsequent BAC biodegradation (Zhang et al., 2011; Hajaya and Pavlostathis, 2013). From the authors' knowledge, only Lambert and Johnston (2001) modeled the BAC inhibition of a specific pathogen, in this case *Staphylococcus aureus*. This work is crucial to understand disinfection with soil contamination, but the model cannot be exploited for optimization since BAC after the treatment is not quantified. However, the work presents interesting observations about the dependence of the disinfection effectiveness with the inoculum concentration that requires further research.

In food microbiology, predictive kinetic models are well established with *ad-hoc* software tools that can be exploited to determine optimal operational conditions, but they are primarily focused on non-chemical (abiotic) disinfection (Geeraerd et al., 2005; Garre et al., 2017). Most works modeling the antimicrobial effect describe the inhibition of microbial growth without considering the antimicrobial kinetics. The exception is the research by Reichart (1994). This work develops kinetic models of microbial inactivation together with the dynamics of molecules responsible for the lethal effect. The theory, however, departs from the standard models on water treatment, instead of using standard modeling approaches in food microbiology.

**Figure 1:**
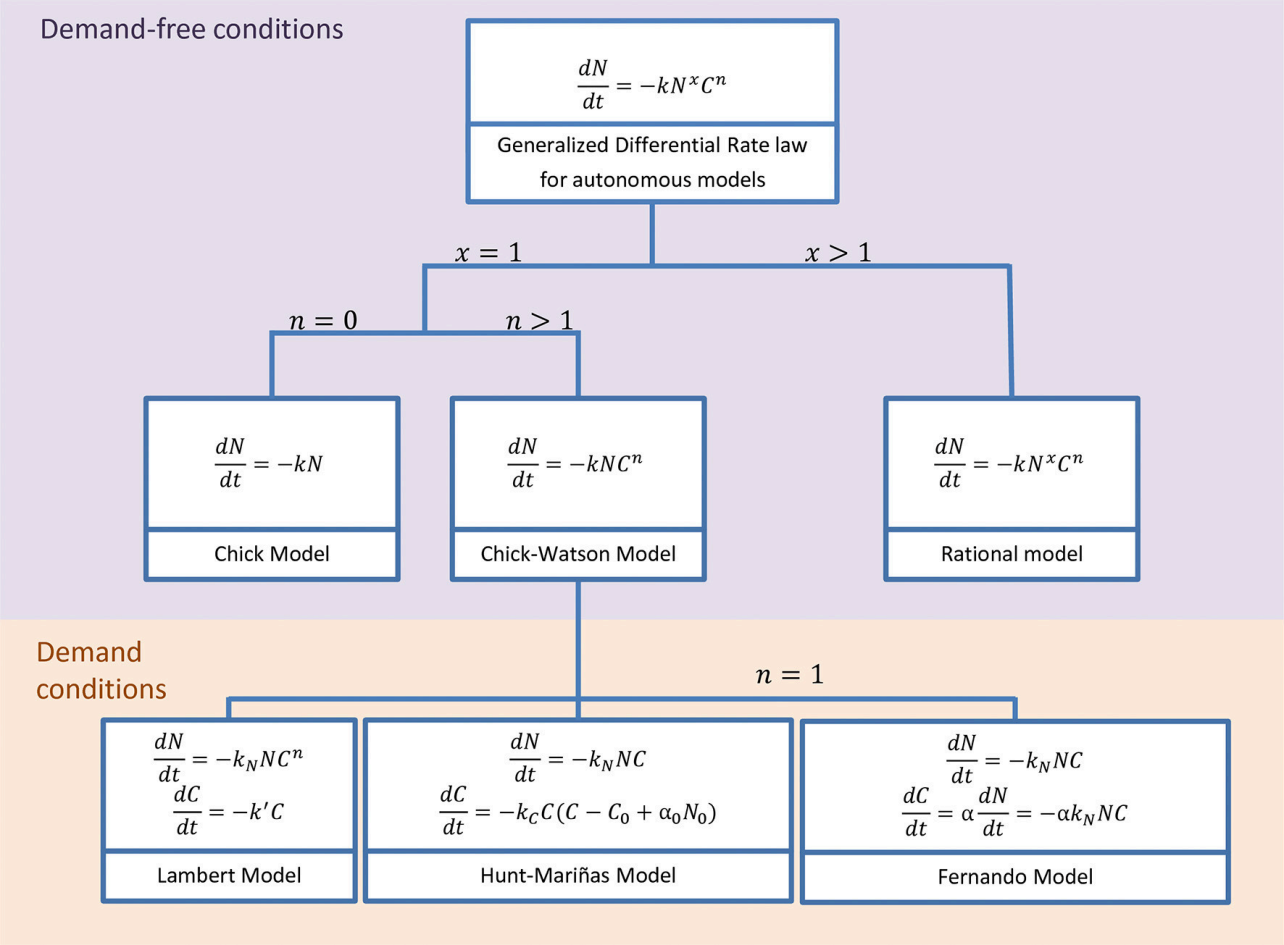
Classical models under disinfectant demand-free conditions assume constant disinfectant concentration and are a special case of the Generalized Differential Rate Law (Gyürék and Finch, 1998; Hunt and Mariñas, 1999). The Integrated form of most of these models can be seen in Gyürék and Finch (1998). Common models using disinfectant demand conditions assume decay independent on the microorganism density: first order decay (Lambert and Johnston, 2000) and second-order rate (Hunt and Mariñas, 1999). Only Fernando (2009) considers that disinfectant decay depends on the microorganism density.

Models considering chemical disinfection are common in water treatment, but disinfectant kinetics are still neglected or too simplistic to study QACs. Most models assume demand-free conditions, that is, the disinfectant is far in excess and remains constant during the treatment. Figure 1 shows a nested model including common autonomous (without an explicit dependence with time in their derivative form) disinfection models under this demand-free condition. They are extremely useful and flexible to model different inactivation curves, but inadequate when the disinfectant is not constant during the treatment, i.e., in demanding conditions. Most disinfectants in water treatment are volatile and therefore the model modification consists of assuming first-order decay kinetics (Lambert and Johnston, 2000). The few exceptions are the model by Hunt and Mariñas (1999) using second-order kinetics and the model by Fernando (2009) assuming that the specific chemical demand (α) depends on the microorganism density during the treatment.

Another drawback that prevents the direct application of water treatment models (Figure 1) is that they assume proportionality between disinfectant and inoculum concentration. They are based on survival or time-kill curves defined in relative terms of log reductions (Ioannou et al., 2007), where the absolute number of active cells is divided by the inoculum concentration. It is therefore implicitly assumed proportionality, i.e., that a 1 log reduction requires the same amount of disinfectant independently of the cell concentration. In other words, if the inoculum doubles, the amount of disinfectant also doubles and therefore the Minimum Inhibitory Concentration (MIC) is proportional to the initial inoculum. However, that contradicts MIC estimations for quaternary ammonium compounds in the literature (Lambert and Johnston, 2001; Ioannou et al., 2007).

This inoculum effect is well-known in antibiotic resistance (Sabath et al., 1975; Thomson and Moland, 2001; Egervärn et al., 2007; Tan et al., 2012; Karslake et al., 2016) with specific descriptions using mathematical models (Udekwu et al., 2009; Bhagunde et al., 2010; Bulitta et al., 2010). Nevertheless, from the author's knowledge, models of the inoculum effect in chemical disinfection are still scarce (Haas and Kaymak, 2003; Kaymak and Haas, 2008) and limited to Lambert and Johnston (2001) for QACs.

In this work, the inoculum effect is studied based on the specific disinfectant uptake, i.e., the mean amount of QACs that is uptaken per cell, that links the pathogen and QAC kinetics. Under the common assumption of proportionality between dose and inoculum concentration, this value is constant with respect to the initial experimental conditions.

The case of study is the optimization *E. coli* inactivation by BAC. The work is organized into three parts: (1) the quantification of the dependence of the specific BAC uptake with respect to the BAC dose and inoculum concentrations; (2) the fit, validation and optimization of the the kinetic model to find the best BAC dose concentration that minimizes the excess of BAC after a treatment and (3) the postulation of possible mechanisms behind the inoculum effect.

## 2. Materials and methods

### 2.1. Experimental materials and methods

#### 2.1.1. Bacterial strain and culturing conditions

*Escherichia coli* CECT 4622 was purchased from the Spanish Type Culture Collection. Stock cultures were kept at −80 °C in Brain Heart Infusion Broth (BHI; Biolife, Milan, Italy) containing 50% glycerol 1:1 (v/v). Work cultures were kept at −20 °C in Trypticase Soy Broth (TSB; Cultimed, Barcelona, Spain) containing 50% glycerol 1:1 (v/v). Before the experiments, 100 μL of work cultures was grown overnight at 37 °C in 5 mL of TSB and subcultured overnight in the same conditions so as to ensure proper growth.

#### 2.1.2. Inoculum preparation

Once activated, the culture was centrifuged (4 min, 8,600g. centrifuge: Sigma, 2-16PK) and the precipitated cells were resuspended in 0.85% (w/v) NaCl. Resuspended cells were adjusted to Abs700 = 0.7±0.001 in 0.85%(w/v) NaCl using a Cecil3000 scanning spectrophotometer (Cecil Instruments, Cambridge, England), corresponding to a concentration of 10^9^CFU/mL. This cell suspension was directly used as inoculum in the experiments or serially diluted in sterile 0.85% NaCl to achieve 10^8^–10^7^CFU/mL the final viable according to the experimental design.

#### 2.1.3. Dynamics of *E. coli* inactivation with benzalkonium chloride

Benzalkonium chloride solutions (Sigma-Aldrich) were prepared in sterile deionized water at three different concentrations for modeling purposes (100, 200, and 300 mg L^−1^) and two more BAC concentrations for the validation experiments (75 and 250 mg L^−1^).

Experimental series were prepared by adding 1 mL of BAC to sterile glass vials containing 1 mL of *E. coli* inoculum and allowed to act for 1, 5, 10, 15, and 20 min at 25 °C without shaking. Negative controls were running in parallel by adding NaCl 0.85% (w/v) instead of BAC.

After each time interval of exposition, 500 μL of the culture were neutralized by adding 500 μL of neutralizing solution (composition L-1:10 mL of a 34 g l-1 *KH*_2_*PO*_4_ buffer (pH7.2); 3 g soybean lecithin; 30 mL Tween 80; 5 g *Na*_2_*S*_2_*O*_3_; 1 g L-histidine) during 10 min at room temperature and used to determine the number of viable cultivable cells. Quantification of viable counts was carried out by serially diluting the bacterial culture and spreading in triplicate onto Trytone Soy Agar (TSA; Cultimed, Barcelona, Spain). Plates were incubated at 37 °C during 24 h and results were expressed as log CFU/mL.

The rest of the culture (1,500 μL was filter sterilized through a 0.2 μm syringe filter (Sartorius, Gottingen, Germany) and the filtrate was used to determine the concentration of BAC outside the cells following the method described by Scott (1968).

### 2.2. Kinetic model and computational methods

#### 2.2.1. Kinetic modeling

The model simulates *E. coli* inactivation and BAC decay kinetics. The inactivation kinetics are based on the generalized Rate Law model by Gyürék and Finch (1998) with *m* = 1. This expression includes most models with demand-free conditions in water treatment (Figure 1). BAC kinetics are modeled assuming that each cell adsorbs a certain quantity α of BAC before dying (see section 3 for details). Therefore the final model tested was:

(1) $$\begin{array}{l} \begin{array}{c} \frac{dN}{dt}=-kN^{x}C^{n}\\ \frac{dC}{dt}=-\alpha kN^{x}C^{n} \end{array} \end{array}$$

where *t* is the contact time in minutes, *N* is the density of viable *E. coli* cells in CFU/ml, *C* is the concentration of *BAC* in ppm, *k* is the inactivation rate constant, *n* the dilution concentration and *x* an empirical constant used in the rational model.

This a deterministic model that can only be applied when the density of viable cells is sufficient to neglect stochastic fluctuations. Stochastic models are scarce in the literature and usually focused on growth dynamics (Augustin et al., 2015; García et al., 2018) or in thermal, but not chemical, inactivation (Nicolaï and Van Impe, 1996). In this work, the dynamics are assumed deterministic by defining a detection limit of 100 cells. This determines the zone where experimental data present large variability and model simulations large uncertainty to be useful. Therefore any value (simulated or experimental) below this limit will be considered as ≤100, without specifying any numerical value.

Integrative forms of these equations are only available for simple cases that can be seen in (Gyürék and Finch, 1998), but not for the case described in this work (BAC decay depends on the inoculum concentration). Therefore the model should be solved using appropriate numerical methods for Ordinary Differential Equations (ODEs).

#### 2.2.2. Computational methods

Different computational methods are required in this work to simulate model in Equation (1), estimate the unknown parameters from available experimental data and optimize the BAC dose concentration as a function of the inoculum numbers. In this work, AMIGO (Advanced Model Identification using Global Optimization) software was used for the simulation and parameter estimation. This is a multi-platform toolbox implemented in Matlab (Balsa-Canto et al., 2016b). The dose optimization was implemented outside of this toolbox but with the same simulation and optimization methods. CVODES (Hindmarsh et al., 2005) was selected to simulate the model and to evaluate the parametric sensitivities. This solver allows us to calculate the confidence intervals of the parameters. For optimization, a global optimizer based on scatter search (eSS, Enhanced Scatter Search) was used (Egea et al., 2010).

Parameter estimation is based on the maximization of the log-likelihood function (LLF). The idea is to find the vector of parameters that gives the highest likelihood to the measured data Balsa-Canto et al. (2016a). For independent measurements with Gaussian noise the problem becomes to minimize the minimum square error weighted with the standard deviations associated with each measurement:

$$J=\sum_{i=1}^{n_{t}}\frac{{\left(\log10(N_{i})-\log10({\hat{N}}_{i})\right)}^{2}}{\sigma_{N}^{2}}+\sum_{i=1}^{n_{t}}\frac{{\left(C_{i}-{\hat{C}}_{i}\right)}^{2}}{\sigma_{C}^{2}}$$

where *N*_*i*_ and *C*_*i*_ are each of the time measurements for *E. coli* and BAC and $\hat{N_{i}}$ and $\hat{C_{i}}$ their respective estimations using model 1 and *n*_*t*_ is the number of time measurements for all experiments. To avoid computational problems derived from the different orders of magnitude a logarithmic scale was used for the viable cells (García et al., 2017b). To solve this optimization problem the standard deviations for *E. coli* (σ_*N*_) and BAC (σ_*C*_) were previously estimated from replicates (2 and 4 replicates for each measurement of viable *E. coli* and one replicate for most of the BAC measurements).

The performance of the estimation is measured using two standard indexes based on mean square errors between model and experimental data for each type of measurements. The first index is the root-mean-square error (RMSE) defined as:

$$\begin{array}{l} RMSE=\sqrt{\left(\frac{1}{n_{t}}\overset{n_{t}}{\underset{i=1}{\sum }}{\left(y_{i}-{\hat{y}}_{i}\right)}^{2}\right)} \end{array}$$

where *y*_*i*_ can be referred to BAC or to *E. coli* viable cells. However, BAC and *E. coli* have different orders of magnitude and for a comparison between their fits the coefficient of variation of the RMSE, CV(RMSE), is preferred:

$$\begin{array}{l} CV\left(RMSE\right)=\frac{RMSD}{\bar{y}} \end{array}$$

where $\bar{y}=\left(1/n_{t}\right)\overset{n_{t}}{\underset{i=1}{\sum }}y_{i}$ is the mean of the data values for each type of measurements.

The confidence intervals for the parameters are estimated by:

$$\begin{array}{l} \pm t_{\alpha /2}^{\gamma }\sqrt{C_{ii}} \end{array}$$

where *C*_*ii*_ are the diagonal elements of the confidence matrix, $t_{\alpha /2}^{\gamma }$ is given by Student's t-distribution with γ the number of degrees of freedom and (1−α)100% selected to 95%. For non-linear system the Cramér-Rao inequality to compute a bound fo the confidence matrix using the Fisher information matrix (Vilas et al., 2018):

$$\begin{array}{l} C\geq F\equiv \text{E}\left\{{\left(\frac{\partial J}{\partial \Theta }\right)}^{T}\left(\frac{\partial J}{\partial \Theta }\right)\right\} \end{array}$$

where Θ is the vector of unknown parameters. Relative confidence intervals (calculated by dividing confidence intervals by the estimated value of the parameter) are also calculated since they are useful when parameters have different orders of magnitude (García et al., 2017a).

To compare the performance among nested models with a different number of parameters, the Akaike Information Criterion (AIC) is used. Its definition using the LFF reads:

$$\begin{array}{l} AIC=2n_{k}-2LLK \end{array}$$

being *n*_*k*_ the number of unknown parameters. The preferred model is the one with the minimum AIC value (Akaike, 1970).

Validations are performed simulating the model for a new set of data or using the cross-validation method. The latter consists of fitting the available data setting aside some experiment or subset of data (Elsner et al., 1994). The obtained model with its estimated parameters is used to predict the data set aside. The process is repeated until all set of data are validated.

## 3. Results and discussion

Modeling allows us to systematically reproduce and optimize complex systems and to motivate new experiments to improve our knowledge. Kinetics of bacterial inactivation are well-known, with several alternatives that are special cases of the generalized model in Figure 1. That is not usually the case for the disinfectant kinetics, and particularly for stable chemicals as QACs. Therefore, next section starts studying BAC uptake at different inoculum and dose concentrations. Section 3.2 focused on the modeling including the description of the mathematical equations, the estimation of the unknown parameters, the assessment of the model predictive capabilities and the optimization of the BAC dose concentrations. Model files, experimental data and scripts to reproduce results can be found in a public repository https://doi.org/10.5281/zenodo.1207616. Finally in section 3.3 the possible mechanisms behind the inoculum effect are discussed and compared with current works in the literature.

### 3.1. Understanding BAC uptake for different inoculum and dose concentrations

#### 3.1.1. BAC disinfection is under demanding conditions

**Figure 2:**
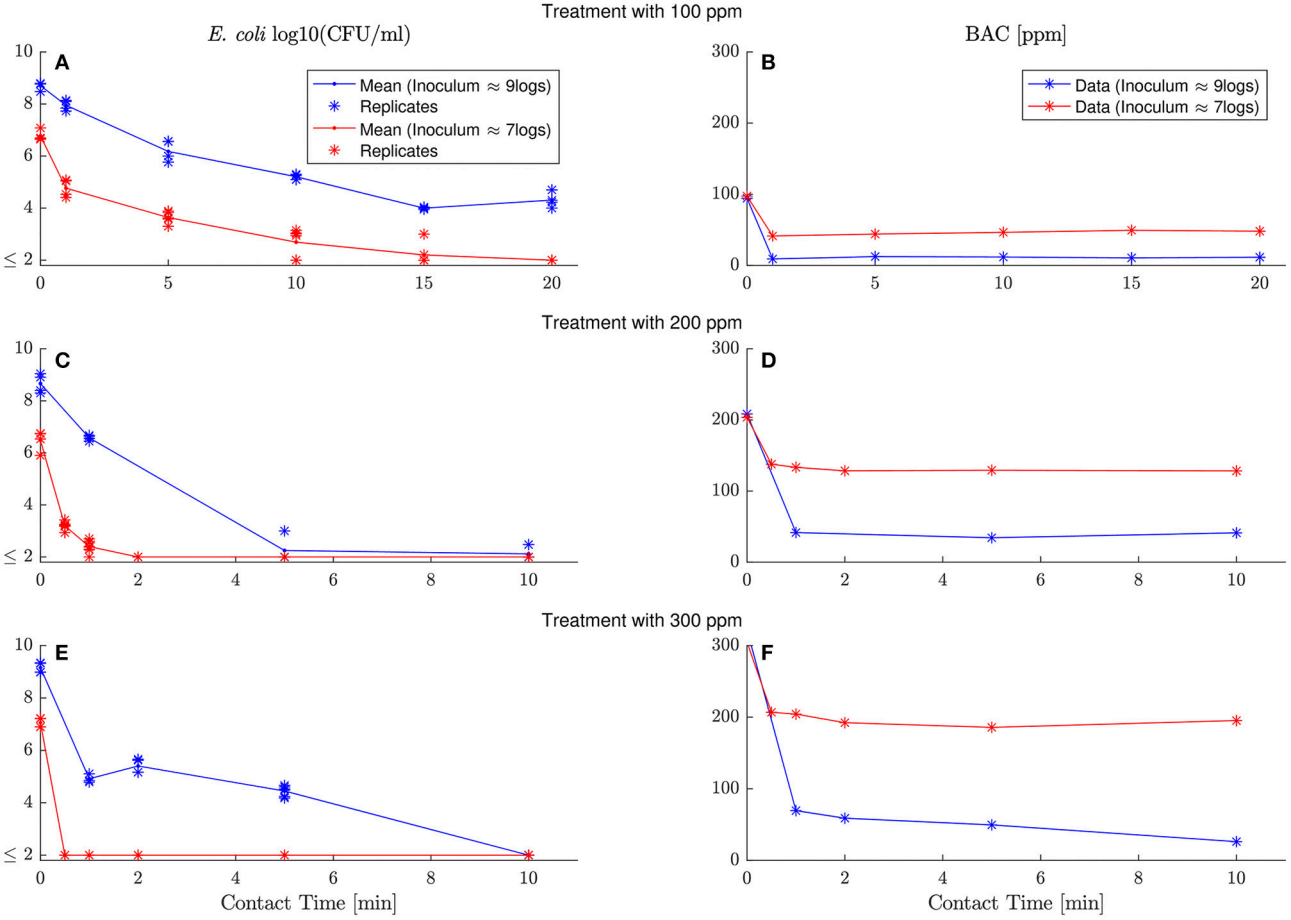
Evolution of *E. coli* viable counts and extracellular BAC concentration with contact time under demanding conditions at different inoculum and dose concentrations. Left and right columns show *E. coli* inactivation and BAC decay, respectively, while each row corresponds to a different dose concentration (100, 200, and 300 ppm). Each panel shows the dynamics with high and low inoculum concentration in, respectively, blue and red. Replicates are represented with asterisks for viable counts, and lines go through their mean values. Detection limit (2 logs) for viable counts is represented with ≤ 2 and gathers all results below this limit. **(A,C,D)** show *E. coli* inactivation by 100, 200 and 300 ppm of initial BAC concentration respectively. **(B–D)** despite BAC decay for the same dose concentration (100, 200 and 300 ppm).

To model the chemical demand of BAC by *E. coli, E. coli* viable cells and extracellular BAC concentration are measured in six experiment with two different inoculum (log_10_(*N*_0_) ≈ 9logs, 7logs) and three different BAC dose concentrations (*C*_0_ = 100, 200, 300 ppm). Figure 2 shows the results arranged in six panels with two columns and three rows. Left and right columns show *E. coli* inactivation and BAC decay, respectively. Different dose concentrations are depicted in different rows. Each panel shows two responses for the two inoculum concentrations tested (blue and red for high and low inoculum concentrations, respectively).

Experiments reveal that BAC is in excess and in demanding conditions for all experiments. After a fast and sharp decay of extracellular BAC, the disinfectant remains constant with values for some cases larger than half the initial dose concentration. Therefore models considering demand-free conditions in Figure 1 are not valid. They assume that the disinfectant extracellular concentration is constant during the process and independent of the bacterial concentrations.

#### 3.1.2. BAC uptake depends on the inoculum concentration and disinfectant dose

Experiments suggest that the BAC residual at final times (C^*^) depends on the dose and inoculum considered. It should be noted that BAC residual is different for each experiment, designed with different inoculum and dose concentrations. Therefore, BAC dynamics cannot be explained using models of first-order decay, so common in water treatment with volatile disinfectants, and a new model has to be proposed.

The objective is to understand how BAC uptake changes as a function of the inoculum concentration and disinfectant dose. The analysis of the disinfectant uptake into the bacterial population is fundamental to understand disinfection in demanding conditions with stable chemicals. Total BAC uptake can be estimated by subtracting the extracellular residual BAC at the end of the experiment C^*^ from the dose concentration applied *C*_0_, i.e., total BAC uptake is $C_{0}-C^{*}$. Here it is assumed that (Assumption 1) BAC extracellular decay is only due to its uptake into *E. coli* cells. This assumption is supported by the observation that volatilization of QACs is negligible and those compounds are not chemically transformed after application (Tezel and Pavlostathis, 2011).

**Figure 3:**
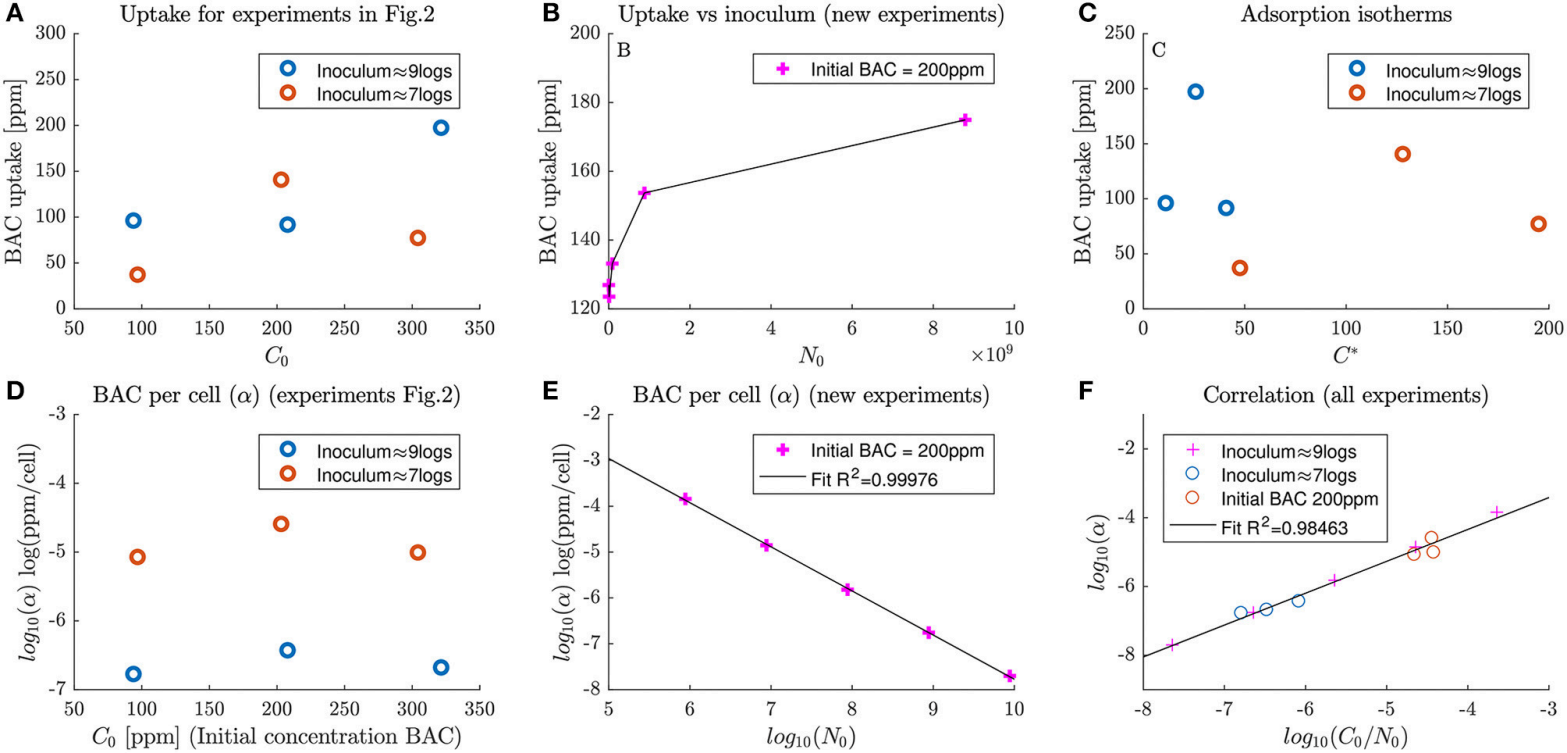
Dependence of BAC uptake and specific BAC uptake (first and second rows of panels respectively) with inoculum size and disinfectant dosage. **(A)** shows the dependence of BAC uptake with dose concentrations at two different inoculum concentrations for experiments in Figure 2. **(B)** depicts the dependence of BAC uptake with the inoculum concentration for a set of new experiments. **(C)** shows the isotherms of uptake for experiments in Figure 2. **(D)** The dependence of specific BAC uptake with dose concentrations at two different inoculum concentrations for experiments in Figure 2. **(E)** depicts the correlation between inoculum and specific BAC uptake for the new experiments at different inocula. Finally **(F)** shows the proposed correlation to explain specific BAC uptake at different inoculum and dose concentrations.

Unfortunately, total BAC uptake does not show a clear trend with respect to the applied dose. Figure 3A shows the calculated BAC uptake with respect to the inoculum concentration. Each circle corresponds to a total uptake of an experiment in Figure 2, and different inoculum concentrations are represented with different colors. The trend is not trivial, cannot be estimated from only three points and it is different for each inoculum concentration.

To detect the dependence of BAC uptake with the inoculum concentration new experiments, depicted in Figure 3B, were designed. Note that in Figure 3A total BAC uptake is larger for larger inoculum concentrations for all cases except for BAC dose of 200 ppm. Results show that total BAC uptake also increases with the inoculum concentration for treatments with 200 ppm. This increase seems to asymptotically approach a saturation level. This trend resembles the one found by Nagai et al. (2003) who estimated extracellular BAC with the Orange II-chloroform method, but for *Pseudomonas fluorescens*.

The use of standard adsorption isotherms to understand BAC uptake was also explored. For both tested inoculum concentrations, as shown in Figure 3C, BAC uptake seems to follow a non-linear trend with a maximum uptake. Those patterns differ from the isotherms found by Ioannou et al. (2007) for BAC and didecyldimethylammonium chloride (a similar quaternary ammonium compound). In the latter work, the uptake increased with the equilibrium concentration, was highly dependent on the chemical used and was analyzed only for low dose concentrations. Probably, the isotherms in this work differ because it was analyzed for high dose concentrations, where there are multiple effects that cannot be cast into an isotherm of adsorption.

With the data available is not possible to find simple trends of total BAC uptake with inoculum and dose concentrations. The following section describes the use of the specific disinfectant uptake as a better descriptor for chemical-demanding conditions than total BAC uptake or BAC isotherms of adsorption.

#### 3.1.3. Specific BAC uptake exhibits a clear trend with respect to the inoculum and dose concentration

Specific BAC uptake (α) is a relative measure of the uptake with respect to the inoculum concentration. Its estimation can be calculated by dividing the total consumption of BAC by the number of cells in the inoculum:

(2) $$\begin{array}{l} \text{specific disinfectant }(\text{BAC})\text{ uptake}=\alpha =\frac{C_{0}-C^{*}}{N_{0}} \end{array}$$

where *N*_0_ is the number of cells in the initial inoculum in CFU per ml. To use this expression it is assumed that (Assumption 2) BAC dose is sufficiently large to kill most of the population. To confirm that the inactivation is complete for the experimental conditions considered in Figure 2, it was verified that there were not viable cells after 24 h for the experiment with the highest inocula and lowest dosage (blue lines in panels Figures 2A,B). A long time after the exposure was considered because BAC is uptaken within the first minutes and cells can become non-viable with some delay.

Making an analogy with a chemical reaction, the specific BAC uptake (α) represents the stoichiometric coefficient, i.e., a constant relating the dynamics of *E. coli* and BAC interplay:

(3) $$\begin{array}{l} N+\alpha C\to \hat{N}C_{\alpha } \end{array}$$

where *N* represents CFU per ml and *C* is the extracellular concentration of BAC. The complex $\hat{N}C_{\alpha }$ symbolizes concentration of non-viable cells.

In the experiments, the specific BAC uptake cannot be constant, unlike in pure chemical reactions, and depends on the inoculum and dose concentrations. If the specific BAC update is constant, the total uptake of BAC would increase linearly with respect to the number of cells in the inoculum. That contradicts the results in Figure 3B. Another way of noting that the specific BAC uptake is not constant is analyzing experiments in Figure 2B. The total uptake of BAC is 80 ppm for an inoculum of 9 logs and 50 ppm for an inoculum of 7 logs. Note that whereas the inoculum size changes two orders of magnitude, total the order of magnitude of BAC uptake remains the same.

The dependence of the specific BAC uptake with respect to the dose concentration for different inocula is depicted in Figure 3D. Specific BAC uptake varies several orders of and, contrary to the total BAC uptake in Figure 3A, follows the same trend for both inocula. The major differences are because of the inoculum size, although there are also changes with the BAC dosage.

As shown in Figure 3E, the specific BAC uptake (α) in logarithmic scale (*log*10(α)) is clearly inversely proportional to the inoculum concentration. Data were the same used in Figure 3B. This means that for low inoculum concentrations, each cell uptakes more BAC than in those experiments with high inoculum concentrations. Note that in all cases the uptake is sufficient to make the cell non-viable.

The information from previous figures can be exploited to calculate specific BAC uptake as a function of the BAC dose and inoculum concentrations. Among the different options tested, the best model consisted assumed a linear dependence of the logarithm of α with respect to $\frac{C_{0}}{N_{0}}$. As shown in Figure 3, this functionality resulted in a *R*^2^ coefficient very close to one.

(4) $$\begin{array}{l} \log_{10}\left(\alpha \right)=a+b\log_{10}\frac{C_{0}}{N_{0}} \end{array}$$

meaning that

(5) $$\begin{array}{l} \alpha =10^{-a}{\left(\frac{C_{0}}{N_{0}}\right)}^{b} \end{array}$$

This model predicts that the specific BAC uptake increases with the dose concentration and decreases with the inoculum concentration. The first observation is expected. Higher concentrations of the disinfectant imply larger concentrations of disinfectant uptake. However, the mechanism by which each cell uptakes less disinfectant when population numbers are large is not obvious. Although it seems to be a common pattern observed for different QACs and bacterial strains as it will be discussed in the last section.

### 3.2. Developing the predictive kinetic model to optimize BAC treatment

#### 3.2.1. The kinetic model reproduces experiments under different dose and inoculum concentrations

Models in chemical disinfection are mostly focused on inactivation kinetics of relevant microorganisms. The generalized differential rate law, in Equation (1), is used to describe the velocity of this reaction. As described in materials and methods, this is a nested model including most of the relevant autonomous models in chemical disinfection.

BAC kinetics are critical to minimize the chemical residuals after a treatment and are calculated from *E. coli* inactivation using the functionality found for the specific BAC uptake in (5). Final dynamic model reads

(6) $$\begin{array}{c} \frac{dN}{dt}=-kN^{x}C^{n}\\ \frac{dC}{dt}=\alpha kN^{x}C^{n}=10^{-a}{\left(\frac{C_{0}}{N_{0}}\right)}^{b}kN^{x}C^{n} \end{array}$$

where the set of unknown parameters is:

$$\begin{array}{l} \theta =\left[a,b,k,x,n\right]. \end{array}$$

Despite there is a rough estimation of *a* and *b* using correlations in previous sections, it is preferable to estimate the whole set of unknown parameters in a single step using all data of *E. coli* and BAC dynamics. This allows us to find the best parameters also to represent the dynamics and mitigates estimation errors due to measurement errors in the residual concentration of BAC (C^*^). For considering that specific BAC uptake is constant, the parameter *b* is fixed to zero being therefore α = 10^−*a*^.

**Figure 4:**
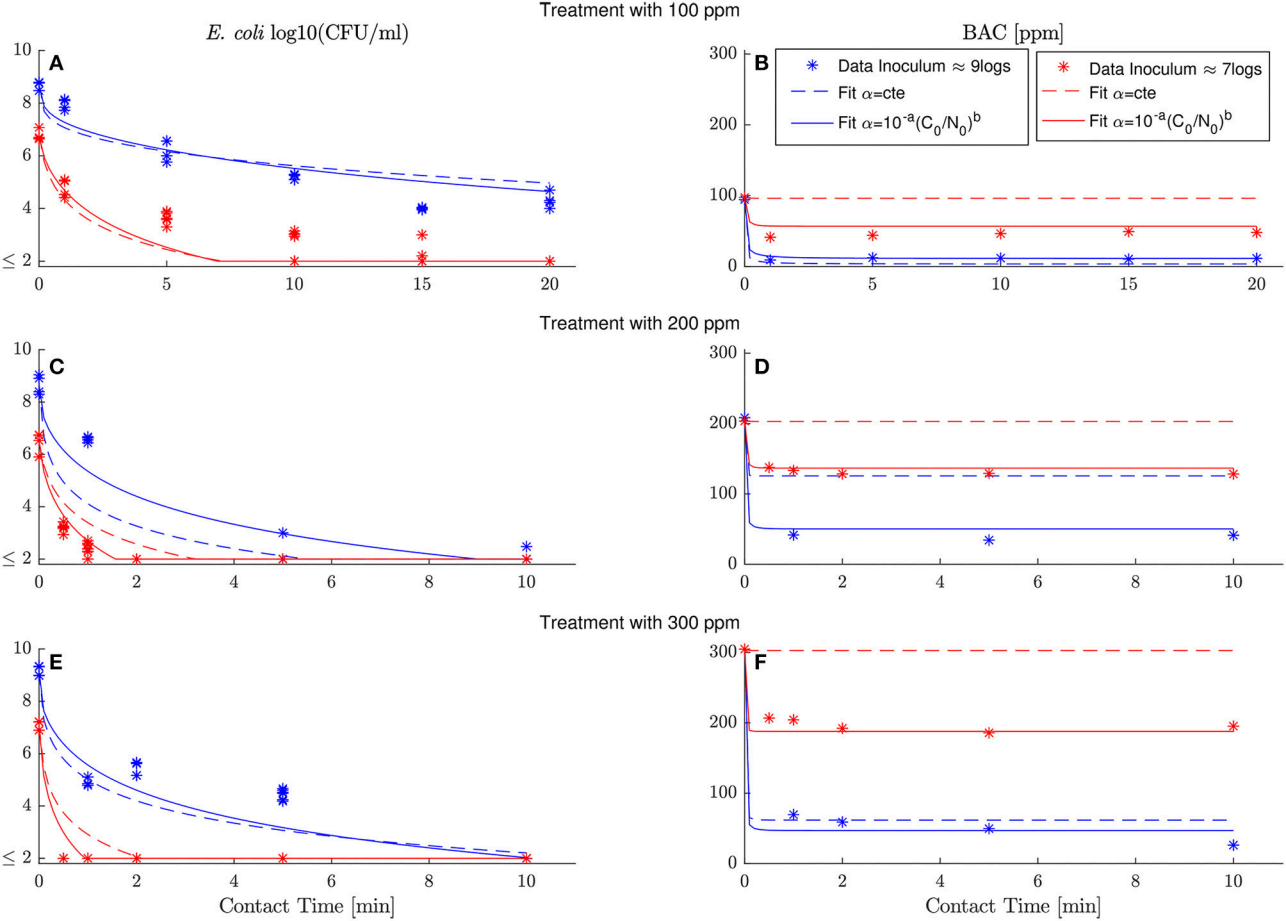
Best fits for model (6) assuming that specific BAC uptake is constant (dashed line) or depends on inoculum and dose concentrations (continuous line). Experimental data marked with asterisks correspond with the results shown in Figure 2. High and low inoculum concentration are shown in blue and red, respectively. The model with specific BAC uptake of the form $\alpha =10^{-a}{\left(C_{0}/N_{0}\right)}^{b}$ fits the data considerably better than the model assuming α = 10^−*a*^ = *cte* with *b* = 0. **(A,C,D)** show model simulations and data of *E. coli* inactivation by 100, 200 and 300 ppm of initial BAC concentration, respectively. **(B–D)** model simulations and data of BAC decay for the same dose concentration (100, 200 and 300 ppm).

Comparisons between experimental and simulated data reveal how critical is to assume a dependence of the specific BAC uptake with the inoculum and dose concentrations. Figure 4 shows the best fit of model (6) assuming that either the specific BAC uptake is constant (dashed line) or depends on the initial experimental conditions (continuous lines). The model reproduces the *E. coli* inactivation kinetics better considering the dependence of specific BAC uptake with the inoculum and dose concentration, but the major differences are in terms of BAC decay. The model assuming that the specific BAC uptake is an invariant stoichiometric coefficient, as in chemical reactions, is not able to reproduce the BAC dynamics for most of the cases, especially for those experiments with low inoculum concentration.

Standard methods, used to quantify the model ability to reproduce the data, confirm that the proposed model outperforms the model with the classical assumption of constant specific uptake. Table 1 shows those methods (see Materials and Methods for details) together with the estimated parameters for both models. The root-mean-square error (RMSE), and its coefficient of variation [CV(RMSE)], quantifies how good the model reproduces the data (fit) in absolute and relative terms, respectively. Note that *E. coli* and BAC measurements are of different nature and of different orders of magnitude. Therefore the CV(RMSE) provides a better description of the goodness of fit. As observed in Figure 4, and expected due to the larger uncertainty in *E. coli* measurements, the model reproduces the data better for BAC than for *E. coli* and always better than the alternative model. The worse fit for the proposed model is for *E. coli* inactivation at low inoculum and low dose since data in this experiment is close to the detection limit for most times, and therefore with the larger uncertainty. Another way to quantify the model performance is the log-likelihood function (LLF) that is maximized to estimate the parameters. For the type of measured error assumed in this work, it is equivalent to the RMSE weighted with the inverse of the square of the standard deviations. Again, the maximum LLF is observed for the model with specific BAC uptake dependent on dose and inoculum concentrations.

**Table 1:** Different criteria to assess the capabilities of both models to reproduce the experimental data.

		**Proposed model (6) with $\alpha =10^{-a}{\left(\frac{C_{0}}{N_{0}}\right)}^{b}$**	**Model (6) with contant α (i.e., *b* = 0)**
Parameters	k	3.75 ± 0.13 (3.41%)	3.22 ± 0.13 (3.86%)
	x	1.25 ± 0.15 (12.00%)	1.34 ± 0.21 (16%)
	n	1.69 ± 0.20 (11.64%)	1.08 ± 0.23 (21.3%)
	a	1.18 ± 0.04 (3.68%)	6.74 ± 0.07 (0.11%)
	b	0.83 ± 0.01 (0.84%)	–
RMSE	*E. coli*	0.54	0.79
	BAC	9.70	60.09
CV(RMSE)	*E. coli*	0.13	0.28
	BAC	0.09	0.57
LLF		−18.09	−42.32
AIC		46.17	94.63

*Unknown parameters are shown as: estimation±confidence interval (relative confidence interval). Root-mean-square error, RMSE, and its coefficient of variation, CV(RMSE), are included for E. coli and BAC. Additionally the performance of both models in terms of the log likelihood function (LLF) and the Akaike criterion (AIC) are shown. Last one allows to compare models with different number of estimated parameters*.

The proposed model reproduces the data without incurring in overparametrization: a usual problem in modeling where fits improve at the expenses of fitting the data noise, outliers and others experimental artifacts (Vilas et al., 2018). To discard overparametrization there are methods considering the model performance as a function of the parameters to be estimated. The Akaike Information Criterion (AIC) is a standard tool to compare nested kinetic models. The proposed model has the minimum Akaike index (Table 1) and the best relative confidence intervals for all parameters. The most uncertain parameters, in relative terms, are the dilution term *n* and the constant of the rational model *x*. That agrees with the observed variability of the data that is much larger for the numbers of bacterial inactivation than for the extracellular BAC concentration. The parameter with more confidence is *b* that models the dependence of the specific BAC uptake with the inoculum and dose concentration. Another evidence of the necessity to consider this dependence.

#### 3.2.2. The model predicts new data

Model-based optimization requires a model with predictive capabilities. Tests in the previous section help us to confirm that the model follows the experimental data used for estimating the unknown parameters (fit). However, for optimization, it is critical to validate if the model with the estimated parameters can predict new data (validation). New data can be inside the rage of the designed experiments for the fit (interpolation), in this work between 7 and 9 logs of inoculum concentration and between 100 and 300 ppm of BAC, or outside (extrapolation). Empirical models commonly predict only interpolation data while mechanistic models or semi-mechanistic models are better reproducing new data outside the range used for the fit.

The proposed model combines mechanistic and empirical arguments. For example, it is based on assumptions 1 and 2 and thanks to this the BAC kinetics are defined. However, the dependence of the specific BAC uptake with the inoculum and dose concentrations is empirical, based on the experimental observations. In this work, the predictive capabilities are tested using the cross-validation method and with two new experiments inside and outside the range of experimental data used for the fit (interpolation and extrapolation).

The experiments in Figure 2, six experiments with three BAC dose and two inoculum concentrations, are used to compute the cross-validation. At each step, five experiments of the six are used for the fit and the remaining one for the validation. In the following step, a different experiment is set aside. After the computation, see Table 2, there are six sets of estimated parameters and six validation experiments. This technique has the advantage of not requiring new experiments and that it helps to identify problems (if there are) with some subset of data.

**Table 2:** Cross validation of final model with experiments in Figure 2.

**Fit for experiments**	**[2,3,4,5,6]**	**[1,3,4,5,6]**	**[1,2,4,5,6]**	**[1,2,3,5,6]**	**[1,2,3,4,6]**	**[1,2,3,4,5]**
k	4.87 ± 0.14	2.94 ± 0.12	4.95 ± 0.12	3.61 ± 0.15	3.64 ± 0.13	3.45 ± 0.18
x	1.23 ± 0.49	1.18 ± 0.15	1.38 ± 0.27	1.25 ± 0.15	1.19 ± 0.16	1.24 ± 0.19
n	2.31 ± 0.53	1.49 ± 0.21	2.01 ± 0.29	1.61 ± 0.22	1.79 ± 0.23	1.56 ± 0.29
a	1.17 ± 0.04	1.24 ± 0.05	1.18 ± 0.04	1.22 ± 0.05	1.12 ± 0.05	1.03 ± 0.06
b	0.83 ± 0.01	0.82 ± 0.01	0.83 ± 0.01	0.83 ± 0.01	0.84 ± 0.01	0.86 ± 0.01
**Validation**	**Exp. 1**	**Exp. 2**	**Exp. 3**	**Exp. 4**	**Exp. 5**	**Exp. 6**
RMSE *E. coli*	1.31	0.74	0.95	0.32	1.08	0.60
RMSE BAC	4.26	11.21	11.90	7.59	15.95	22.06
CV(RMSE) *E. coli*	0.22	0.20	0.19	0.11	0.21	0.21
CV(RMSE) BAC	0.17	0.21	0.15	0.05	0.15	0.10

*This includes experiments at different BAC dose concentrations: 100 ppm (1 and 2), 200 ppm (3 and 4), and 300 ppm (5 and 6) and with two inoculum concentrations (odd and even numbers correspond to high and low inoculum concentrations, respectively). Table shows the six sets of estimated parameters removing one different experiment each time that is used for the validation. The indexes RMSE and CV(RMSE) are used for analyzing the performance of the validations*.

Estimated parameters (and confidence intervals) for the cross-validation are similar to the those obtained fitting the six experiments. Larger deviations with respect to the estimated parameters in Table 1 are, as expected, for extrapolation experiments, like experiment 1 with the lower dose concentration (100 ppm) and high inoculum concentration (9 logs).

The validation experiments of the cross-validation confirm that the proposed model has good prediction capabilities. RMSE and CV(RMSE) are similar and lower for the interpolation experiments (3 and 4). Worse validations are for experiment 1 in terms of *E. coli* CV(RMSE) and experiment 2 for BAC CV(RMSE). But even for those experiments, their kinetics are not so far from the experimental data as shown in the Figure S1 in Supplemental Data. It should be stressed that validation experiments are obtained with estimated parameters from other experiments, and therefore their behavior, as well as the RMSE and CV(RMSE), are rarely better than same indexes for the fit and should be carefully compared with results in Table 1.

Two additional experiments are carried out to further test the predictive capabilities and weakness of the model. The interpolation consists on a concentration similar to 8 logs for the inoculum and 250 ppm for the BAC dose. The extrapolation experiment was designed with 8 logs for inoculum and 75 ppm for BAC dose concentration.

**Figure 5:**
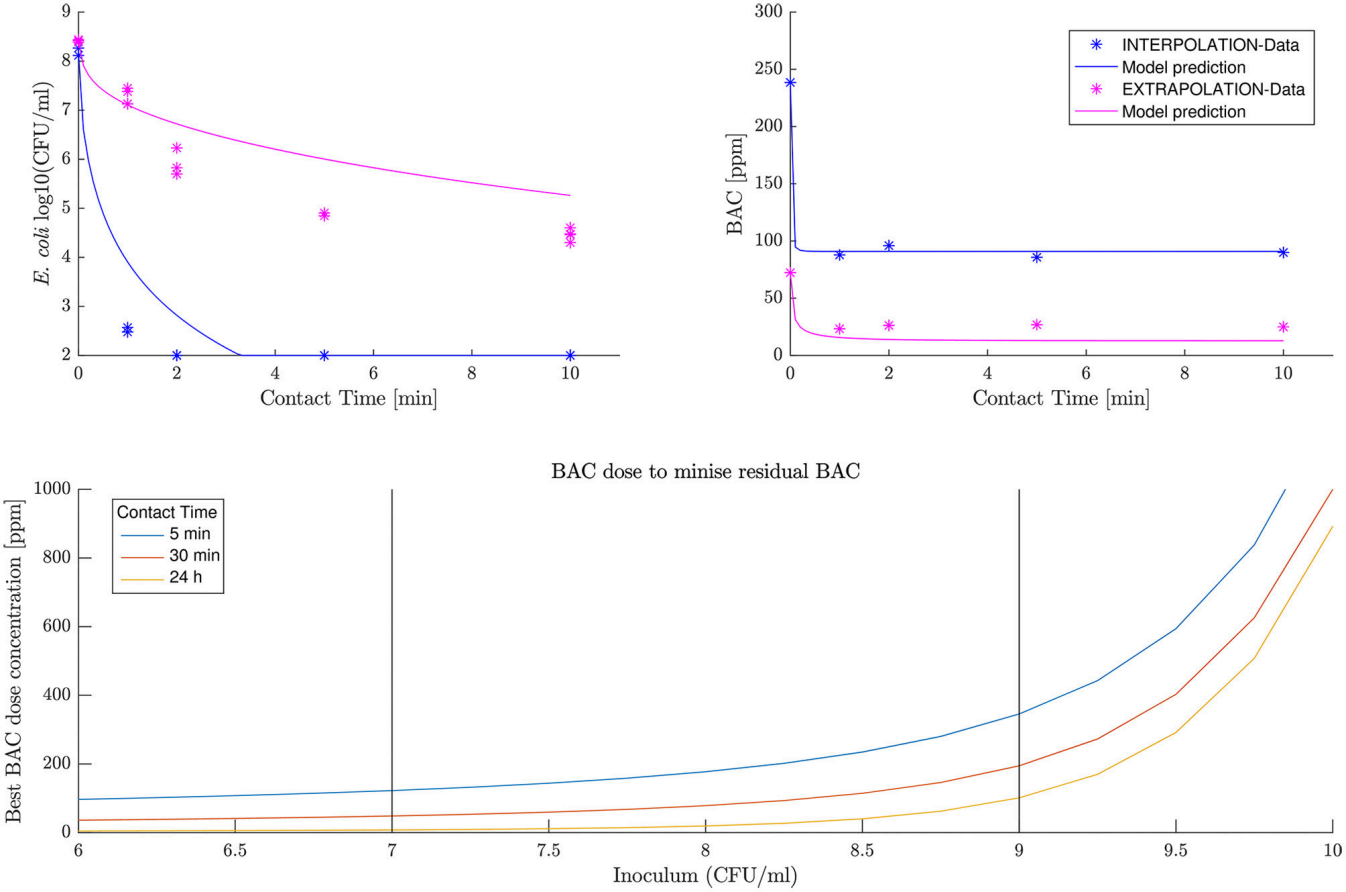
New data prediction (first row) and optimal BAC dose concentration (second row).

The model predicts both experiments, especially for BAC decay and interpolation data. First row in Figure 5 shows the predicted BAC and *E. coli* kinetics. Results are considered satisfactory since BAC decay is predicted particularly good. It should be noted that BAC dynamics are more relevant in the context of this work as for the dose concentrations used a complete inactivation of *E. coli* is assumed, and the prediction always overestimates the *E. coli* numbers being in the safest scenario.

#### 3.2.3. The model allows to optimize dose concentration

Model-based optimization also requires, in addition to a predictive model, that the estimated parameters do not change with the experimental conditions. That is a common problem with other models in the literature fitting the data separately for each experiment. Those models also require more data to assume good confidence intervals of the parameter.

For the proposed model, the parameters are the same independently on the inoculum and dose concentration and can be used to optimize doses for a given inoculum. The formal description of the problem is as follows:

(7) $$\begin{array}{ll} \underset{C_{0}}{\min} & {\left(C^{*}\right)}^{2} \end{array}$$

(8) $$\begin{array}{ll} \text{subject to} & N^{*}<=100\;(\text{detection limit}) \end{array}$$

where C^*^ and N^*^ is the BAC and *E. coli* numbers at the final time and *C*_0_ is the dose concentration. A final number of *E. coli* less than 100 is required for being the detection limit of the data and knowing that the model tends to overestimate this number, but other criteria can be easily selected. Three scenarios are considered: 5, 30 min, and 24 h to illustrate how optimal treatments change with contact time.

The second-row panel in Figure 5 shows the minimum BAC dose to reduce the population to 100 viable cells for different inoculum concentrations. The black vertical bars define the range of data used for the fit, and therefore where the model confidence is greater. Data shows how the dose increases exponentially with the inoculum concentration. Probably this is an overestimation of the BAC dose required for inoculum concentrations greater than 9 logs where the model has not been tested.

### 3.3. Discussing the mechanisms in BAC disinfection and the relevance of quantifying the inoculum effect

#### 3.3.1. Mechanisms behind the specific BAC uptake

Salton's theory, from the sixties, is still the reference when studying the mechanisms behind QACs disinfection (Salton, 1968). This work proposed the following series of events: (i) QAC adsorption to and penetration of the cell wall; (ii) reaction with the cytoplasmic membrane followed by membrane disorganization; (iii) leakage of intracellular lower-weight material; (iv) degradation of proteins and nucleic acids; and (v) cell wall lysis caused by autolytic enzymes.

In the context of Salton's theory, the specific BAC uptake quantified in this work represents the equilibrium between the influx (BAC adsorption or penetration) and the efflux (BAC leaking or cell wall lysis).

It should be stressed that, if efflux is relevant, it occurs at the time scales of the influx and leakage seems to be the main mechanism. Extracellular BAC quickly decays in the experiments within first minutes (Figure 4), suggesting that the equilibrium between influx and efflux is fast. On the other hand, attending to some observations while setting-up the methodology to measure extracellular BAC, leakage seems more relevant than cell lysis. Even for high BAC doses, extracellular BAC concentration was substantially larger when cells were separated by centrifugation than by filtering. Since cells are supporting a major pressure with centrifugation, this may indicate that cell membrane retains the BAC (without lysis) and the intracellular material only leaks when sufficient pressure is applied.

However, BAC leakage cannot be fully understood with the available model and data. Overparametrized models have been obtained when trying to expand the model to explicitly consider the influx and efflux of BAC. For a proper definition of the leakage, it is critical to measure another representative variable of this mechanism. A possibility, to be considered for future works, could be to measure an energy-dependent variable linked to efflux pumps, such as in Nagai et al. (2003).

#### 3.3.2. Mechanisms behind the inoculum concentration

The main difficulty to understand the inoculum effect is that it depends on the microbial species and strain and on the antimicrobial type and compound (Udekwu et al., 2009; Karslake et al., 2016).

The effect is critical in drug treatments, where infections exceeding a critical inoculum concentration survive otherwise effective treatments. Works studying antibiotic susceptibility usually postulate that the medium is modulated by the number of bacteria in the population. For example, Karslake et al. (2016) proposed a mechanism based on pH media changes and Datta and Benjamin (1999) in fluctuations of the medium acidity. Other mechanisms such as a decrease in per-cell antibiotic concentration are also being proposed (Udekwu et al., 2009).

Nevertheless, only a few works, from the author's knowledge, have quantified the inoculum effect with BAC (Lambert and Johnston, 2001; Ioannou et al., 2007). This research found that the relationship between the required disinfectant dose and the inoculum level, such as with antibiotics and in this work, was not proportional.

Lambert and Johnston (2001) quantified the inoculum effect when studying inactivation of *Staphylococcus aureus* with BAC using the fractional area. The work found that BAC dose has to be inversely proportional to the inoculum concentration to the power of 0.44. For a similar expression, a value of 0.83 is estimated in this work. It may be speculated that the discrepancy is due to the differences between *E. coli* and *Staphylococcus aureus* or because cell inactivation is measured with different methodologies (viable cells and optical density). Moreover, the proposed model in this work includes other effects, such as the dose concentration, that may cause the differences. In any case, a similar trend, and therefore quantification, for the inoculum effect was observed in both cases.

On the other hand, Ioannou et al. (2007) proposes the use of the adsorption isotherms to quantify this effect for *Staphylococcus aureus*. Extracellular BAC decay seems too fast for an energy-dependent mechanism and Ioannou et al. (2007) assumed that uptake is mainly because of adsorption. Whereas their results resemble a Langmuir isotherm, data in this work fits better to a C-shaped isotherm (Figure 3C). The differences may be again attributed to the microbial species considered. However, it should be also stressed that the BAC doses used in this work were more aggressive than in Ioannou et al. (2007) and therefore other mechanisms could become relevant, such as BAC penetration and leakage, in addition to adsorption.

In fact, experiments in this work using high concentrations of BAC indicates that the specific BAC uptake could be a better descriptor than adsorption isotherms. Note that both are related, but specific BAC uptake may better incorporate other mechanisms that are relevant at significant concentrations of the disinfectant and in antibiotic treatments, such as leakage or pump efflux. Moreover, it directly links the interplay between disinfectant and bacterial inactivation in a simple matter for modeling that can be used as an analogy of a biochemical reaction (3).

It should also be mentioned the theoretical work by Fernando (2009), who also uses the concept of specific disinfectant update (α). However, it assumes that this quantity may change during the treatment because the cell may become less susceptible to the chemical agent. In that case, BAC uptake per cell cannot be calculated using (2). An alternative model was tested (data not shown) assuming a similar dependence of α with viable cells. Good fits were obtained for this case but without improvements. Probably the disinfection process is too fast to observe any change in susceptibility or resistance during the treatment. Also, dependence on initial inoculum, instead of viable cells, has more sense as disinfectant can be adsorbed or enter the cells even if cells are not viable.

Experiments in this work may suggest that the observed differences in specific BAC uptake (or adsorption) are because cells aggregate forming clusters for dense populations, and therefore less membrane surface is exposed to BAC. That would explain results (Figure 3B) showing that the specific BAC uptake (BAC uptake per cell) decreased with the number of cells. To test the hypothesis a population of 8 log bacteria was observed by acquiring phase-contrast images, see the Figure S2 in Supplemental Data. Unfortunately, only cells while and just after division where attached to each other. Therefore the inoculum effect cannot be the attributed to a decrease of membrane exposure for dense populations.

Quorum sensing could be also a plausible explanation of the observed inoculum effect. Quorum sensing circuits regulate gene expression through extracellular signal molecules proportional to the cell density (Miller and Bassler, 2001). Therefore, different inoculum sizes obtained from a stationary phase culture have different concentrations of signal molecules (autoinducers), and different behavior of the bacterial population. In this work, specific BAC uptake changes with the inoculum. This uptake may depend on multidrug resistance efflux pumps, for example, that are regulated by quorum sensing in several bacterial genera, such as *P. aeruginosa* (Köhler et al., 2001) and *E. coli* (Yang et al., 2006).

## 4. Concluding remarks

In this work, the inoculum effect for *E. coli* inactivation by BAC has been quantified. The equation describing the effect is combined with a kinetic model of BAC and *E. coli* to determine the treatment that inactivates most of the population with a minimum dose concentration. Interestingly this optimum dose is not proportional to the inoculum concentration, but it increases exponentially (Figure 5). The reason behind is that each cell uptakes less BAC when the inoculum concentration increases. Possible mechanisms are discussed, but more research is needed.

The predictive model of the characteristics here developed allows analyzing different effects, such as the contact time. It may also set the basis to develop a theory for the inoculum effect applicable to other pairs of bacteria-antimicrobials. This will, ultimately, guide the search for the relevant causes of this effect.

## Author contributions

MG designed the theoretical study, conducted the computational experiments and drafted the manuscript. MC designed the experimental study and analyzed the data. All authors approved the final version of the manuscript.

### Conflict of interest statement

The authors declare that the research was conducted in the absence of any commercial or financial relationships that could be construed as a potential conflict of interest.

## Supplementary material

Figure S1 Capabilities of the model to predict dynamics inside the range considered during calibration. Each of the dynamics is calculated from the estimated parameters (Table 2) by calibrating the other experiments. The model is able to predict situations not considered during calibration but in the range of the experiments considered for the calibration

Figure S2 Phase-contrast images of a population of *E. coli* with a density of 8 logs per milliliter.

### Supplemental data

#### Cross-validation

The model cross-validation is shown in Figure S1. Note that results are validation experiments, i.e., the parameters to simulate the kinetics were determined without considering this set of experimental data.

#### Code to reproduce the computational results

Model files, experimental data and scripts to reproduce results can be found in a public repository https://doi.org/10.5281/zenodo.1207616.

#### Phase-contrast image of a 8 log population

To test if aggregation is a plausible explanation for the inoculum effect, a population of 8logs was observed using a DMX1200 camera mounted on an Eclipse TE2000-S inverted microscope, Nikon Japan, with a 40×/0.75 NA objective.
