# Supplementary figures and images for: Optimization of E. coli Inactivation by Benzalkonium Chloride Reveals the Importance of Quantifying the Inoculum Effect on Chemical Disinfection
Source: Front Microbiol. 2018 Jun 26;9:1259. doi: 10.3389/fmicb.2018.01259 (PMC6028699; doi:10.3389/fmicb.2018.01259)

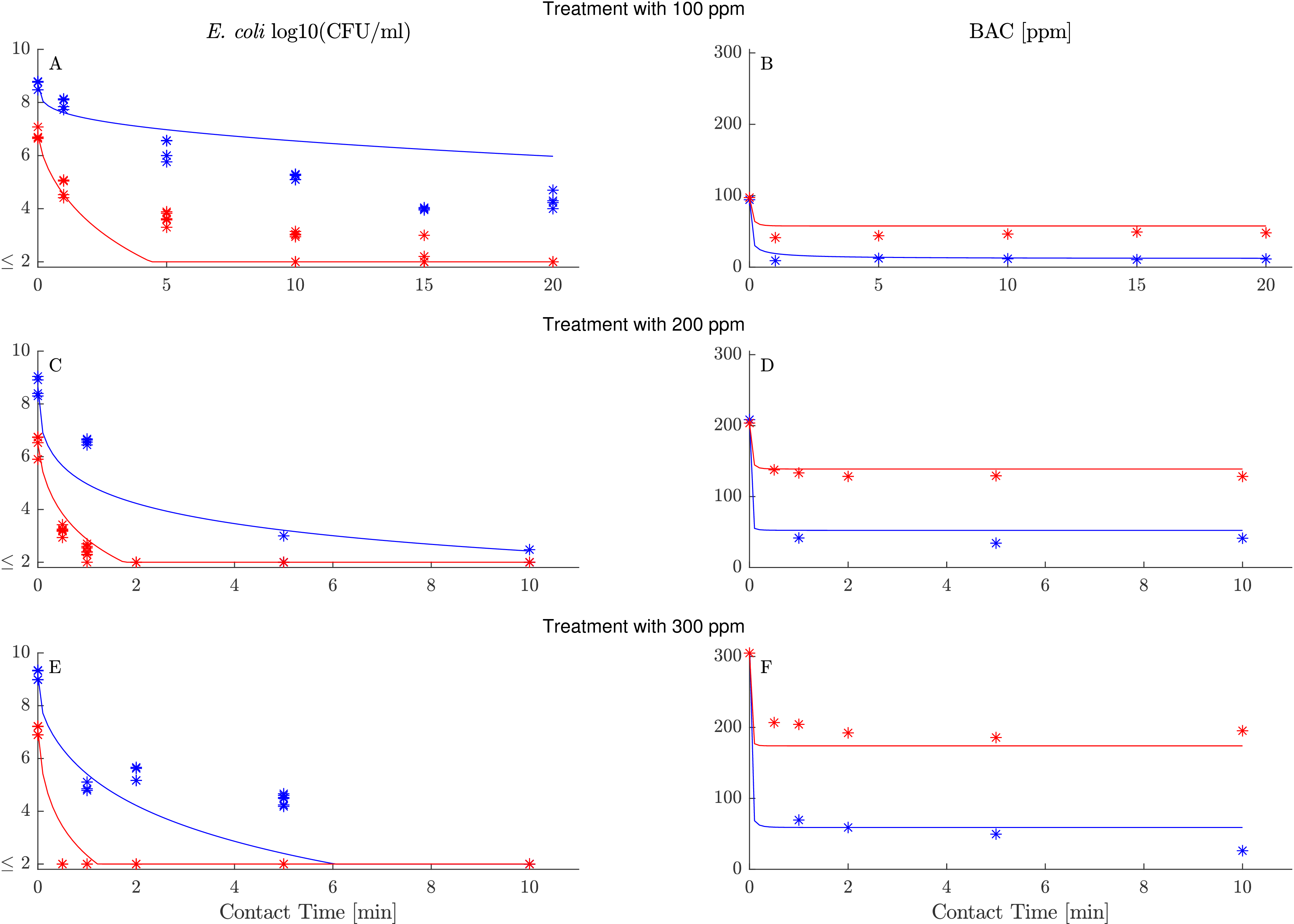

Supplement: Figure S1 — Capabilities of the model to predict dynamics inside the range considered during calibration. Each of the dynamics is calculated from the estimated parameters (Table 2) by calibrating the other experiments. The model is able to predict situations not considered during calibration but in the range of the experiments considered for the calibration [file Image_1.tiff]

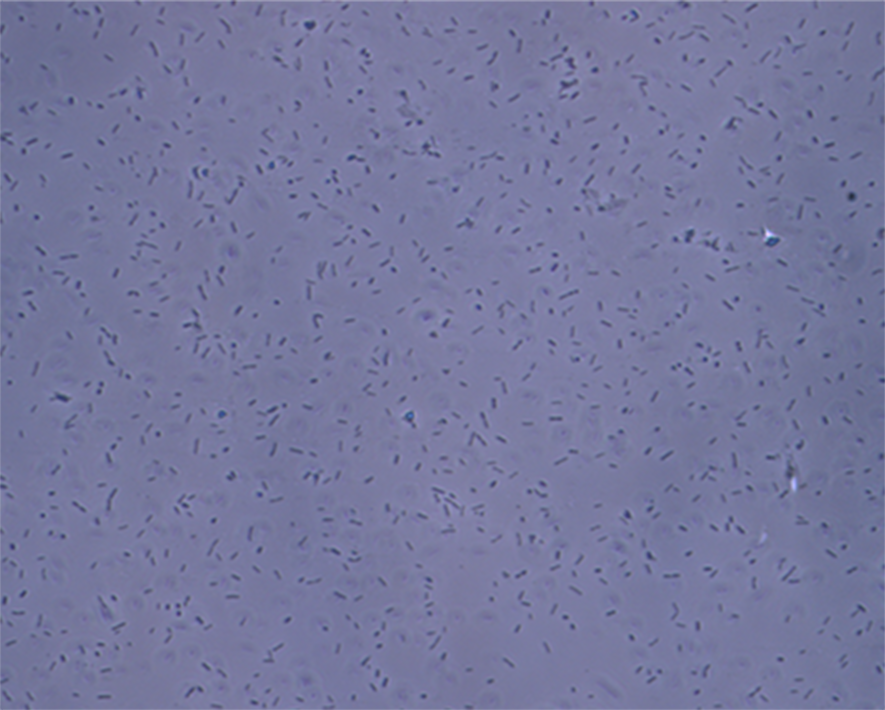

Supplement: Figure S2 — Phase-contrast images of a population of E. coli with a density of 8 logs per milliliter. [file Image_2.tiff]
